# A New Porcelain Crown

**Published:** 1905-06-15

**Authors:** C. H. Worboys


### A NEW PORCELAIN CROWN.

C. H. WORBOYS, D.D.S.

Presented at the meeting of the Southwestern Michigan Dental Society.

I will describe what to me is a new method of adapting a porcelain crown, or hood, to a natural tooth which does not require a knowledge of porcelain technique or that the pulp be devitalized and removed.

The tooth that is to be operated upon should first be anesthetized with cocain and then the enamel ground off, leaving a shoulder just below the gum line and the stump conical in form, if practicable, leaving sufficient dentine to protect the pulp.

The tooth that is used for this operation may be a countersunk pin tooth, such as is used for rubber plates, or a Davis crown, or any other porcelain tooth, or crown that has a hole in its center, the larger the better, usually.

An impression is taken of the prepared stump with white sheet gutta-percha with a tray made of a band of German silver, or copper, shaped to fit the gum on the labial and lingual sides. For this purpose I have found that a shell, such as is used for a seamless crown, that can be placed between the adjoining teeth, to be very convenient.

The gutta-percha is warmed well, then placed in the tray, carried firmly to place, and held until it cools. It must be carefully removed, then a piece of the sheet of the gutta-percha, about three-fourths of an inch wide, is warmed and wrapped around the impression and tray, the edges being stuck together. The impression is then poured full of any of the low-fusing alloys, producing a model of the stump on which to roughly fit the crown, which is done entirely with grinding stones. For the grinding I like the carborundum stones mounted with shellac. The gem stones are necessary to grind the inside of the crown so that it will fit over the stump.

By painting the metal model with oxide of iron and glycerine, you can tell where the crown strikes on the model, and by frequently trying on the crown and grinding carefully it can be made to fit very closely, occasionally trying in the mouth for noting correct position.

The final fitting should be done in the mouth where the stump is to be painted and the crown ground, or the crown painted and the stump ground as you prefer for the best interests of the case.

When the crown is fitted and ready to set, the stump should be cleaned and dried, then a quick-setting inlay cement is mixed thin and placed in the crown and the crown placed as soon as possible.

